# Computational and Cytotoxicity Evaluation of Phyllanthus Urinaria-Derived Compounds as Potential Anti-Cervical Cancer Agents via HPV-16 E6 Oncoprotein Inhibition

**DOI:** 10.3390/ijms27114780

**Published:** 2026-05-26

**Authors:** Andi Darma Putra, Safika Safika, Fadilah Fadilah, Kartiwa Hadi Nuryanto, Aldi Tamara Rahman, Lasmini Syariatin, Naufal Syafiq Darmawan, Kevin Nathaniel Cuandra, Firda Puspita, Gatot Purwoto

**Affiliations:** 1 Division of Gynecology-Oncology, Department of Obstetrics and Gynecology, Faculty of Medicine, Universitas Indonesia, Cipto Mangunkusumo Hospital, Central Jakarta 10430, Indonesia; kartiwa_h_nuryanto@yahoo.com (K.H.N.); gatotpurwoto@gmail.com (G.P.); 2 Ovarian, Tubal, and Peritoneal Malignancy Research Unit, Department of Obstetrics and Gynecology, Faculty of Medicine, Universitas Indonesia, Cipto Mangunkusumo Hospital, Central Jakarta 10430, Indonesia; 2010422018_aldi@student.unand.ac.id (A.T.R.); lasminisyariatin@mail.ugm.ac.id (L.S.); naufal.syafiq71@ui.ac.id (N.S.D.); itsfirdapuspita@gmail.com (F.P.); 3 Dopamine Science Institute, Depok 16431, Indonesia; 4 School of Veterinary Medicine and Biomedical Sciences, IPB University, Bogor 16680, Indonesia; safika@apps.ipb.ac.id; 5 Department of Medicinal Chemistry, Faculty of Medicine, Universitas Indonesia, Central Jakarta 10430, Indonesia; fadilah.msi@ui.ac.id; 6 Department of Medicine, Faculty of Medicine, Andalas University, Padang 25175, Indonesia; 2010317016_kevin@student.unand.ac.id

**Keywords:** cervical cancer, HPV-16, *Phyllanthus urinaria*, molecular docking, cytotoxicity assay

## Abstract

Cervical cancer remains one of the most lethal cancers affecting women, with infection by high-risk Human Papillomavirus (HR-HPV), especially HPV-16, recognized as a primary cause. *Phyllanthus urinaria*, a plant that grows in Indonesia, has demonstrated notable both antiviral and anticancer properties. This study aimed to investigate the potential of *P. urinaria* as both an antiviral and anti-cervical cancer agent. The HPV-16 E6 protein was modeled using homology modelling, with model accuracy verified through torsional angle assessment and identification of conserved regions. Molecular docking was performed to examine E6–p53 interactions. Fraction of n-hexane compounds of *P. urinaria* were further evaluated for their interaction with E6 by molecular docking and molecular dynamics simulation. Additionally, in vitro cytotoxicity assays were conducted using HaCaT (normal keratinocyte) and HeLa (cervical cancer) cell lines. Compounds from *P. urinaria* were found to interact with E6 within conserved regions and these interactions were more stable conformationally than those observed for p53. In vitro assay demonstrated that *P. urinaria* exhibited moderate cytotoxicity against HeLa cells but had limited toxicity toward HaCaT cells. The n-hexane fraction of *P. urinaria* leaves exhibits anti-cervical cancer activity by inhibiting HPV-16 E6 and eliminating cervical cancer cells.

## 1. Introduction

Cervical cancer ranks as the second most common cancer and a leading cause of mortality among women of reproductive age, particularly in countries with a low Human Development Index (HDI), such as those in Central African and South Asia regions [1]. A primary contributor to this disease is infection with high-risk human papillomavirus (HR-HPV), with HPV-16 being responsible for approximately 45.5% of cervical cancer cases worldwide and as many as 60% of cases in Indonesia [2]. HPV is a double-stranded DNA virus with a genome of 7200–8000 base pairs, divided into three regions. One critical region is the Early Protein Open Reading Frame (ORF) that encodes the E6 protein and facilitates viral genome persistence through the binding and degradation of essential tumor suppressors, such as p53 [3]. This region further plays a pivotal role in cervical cancer progression and immortalization [4]. Current management of cervical cancer relies on a multimodal strategy, incorporating surgery, chemotherapy, and radiotherapy. However, these traditional approaches are often accompanied by significant side effects, such as gastrointestinal disturbances, skin reactions, sexual function disruptions, anemia, alopecia, and drug resistance [5,6,7]. Consequently, overall 5-year survival rates have not shown substantial improvement over the past 25 years [8,9]. This urgent clinical reality underscores the need for innovative and safer therapeutic approaches. A promising strategy involves the use of medicinal plants to target the E6 oncoprotein, which is continuously expressed in infected cells and is essential in cervical carcinogenesis, thus providing a potential solution to the limitations of current therapies.

Indonesia is the second largest country globally with the highest biodiversity, offering a remarkable source of structurally diverse natural compounds that hold promise as drug candidates [10]. Diverse native species have historically been recognized as sources of pharmacologically active chemicals, including polyphenols, flavonoids, quinones, and saponins, which exhibit a variety of bioactivities such as antioxidant, anti-inflammatory, and anticancer properties [11,12]. *Phyllanthus urinaria*, as a native medicinal plant, has demonstrated notable biological activities, including antiviral and anticancer effects, while showing a favorable safety profile on normal cells [13]. Chen et al. (2023) showed that ethyl acetate extract of *P. urinaria* possessed the ability to generate reactive oxygen species (ROS) to suppress hepatitis B virus [14]. Previous studies employing extracts of *P. urinaria*, which contain polyphenols, flavonoids, and tannins including gallic acid, quercetin, corilagin, and geraniin, have also demonstrated anticancer activity toward osteosarcoma, lung cancers and prostate cancers by inducing apoptosis via the inhibition of MAPK/ERK and AKT signaling pathways [15,16,17]. Despite its established bioactivity, the mechanistic potential of *P. urinaria* in HPV-associated cervical cancer has yet to be investigated, particularly with its ability to modulate HPV oncogenic processes. Therefore, a focused investigation is needed to evaluate the ability of this plant to simultaneously inhibit HPV-driven oncogenesis and induce cancer cell death, employing both in silico and in vitro approaches to illuminate its value in cervical cancer therapy.

In this study, we aim to explore bioactive compounds derived from *P. urinaria* that have the ability to target the HPV-16 E6 oncoprotein using computational analysis. This study employed a target-guided screening approach that focuses on non-polar compounds obtained through n-hexane extraction with potential affinity for the HPV-16 E6 oncoprotein, in contrast with previous research that mainly evaluated crude or polar extracts for standard cytotoxicity. n-Hexane extraction selectively isolates lipophilic compounds, such as triterpenoids and steroids, which are associated with enhanced membrane permeability and anticancer properties [18,19]. Subsequently, we verified their anticancer efficacy and biocompatibility via in vitro assessment on both normal and cervical cancer cells. This research aims to establish a foundation for natural-product-based interventions targeting HPV-induced cancers, with future initiatives directed towards preclinical development, combination analysis with current therapies, and the potential incorporation of promising candidates into personalized treatment strategies.

## 2. Results

### 2.1. Metabolite Profile of the n-Hexane Fraction of P. urinaria

**Figure 1:**
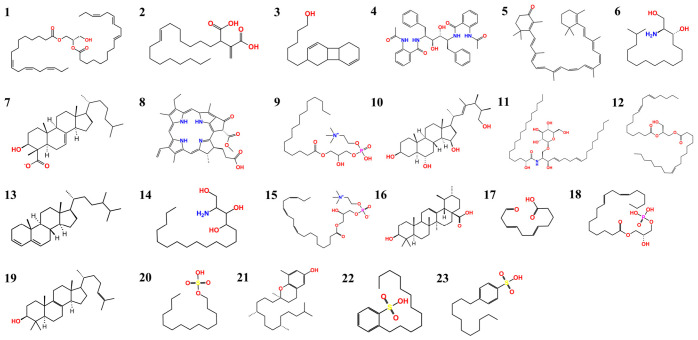
Chemical structures of putatively identified metabolites from *P. urinaria* leaves based on LC–HRMS analysis. Compound numbers correspond to those listed in Table 1.

LC–HRMS analysis facilitated the putative characterization of numerous metabolite features within the examined extract. Supplementary Figures S1 and S2 presents total ion chromatogram (TIC) and representative extracted ion chromatograms (EICs) that demonstrate the chromatographic behavior and peak resolution of selected compounds, respectively. Annotations of putative compounds, derived from accurate mass matching and hierarchical database searching, are presented in Supplementary Figure S3 and Table S1, while compound annotations based on MS/MS (MS^2^) fragmentation analysis are provided in Supplementary Figure S4. Based on these analytical results, a total of 23 major compounds were associated in the leaves of *P. urinaria*, as presented in Table 1 and Figure 1. The metabolite profile primarily consisted of lipid derivatives such as glycerolipids, glycerophospholipids, fatty acyls, prenol lipids, sphingolipids, and sterols. Lipid-based compounds exhibit several advantages, including enhanced permeability across cell membranes, which facilitates efficient cellular delivery and allows for the targeting of specific proteins involved in cancer cell signaling [20]. Lipid derivatives are essential in viral fusion, entry, and the inhibition of replication at the cell plasma membrane [21]. Thus, the compounds present in *P. urinaria* exhibit potential dual functions as antiviral and anticancer agents, necessitating further research.

### 2.2. E6 HPV-16 Conserved Region Homology Modelling

**Figure 2:**
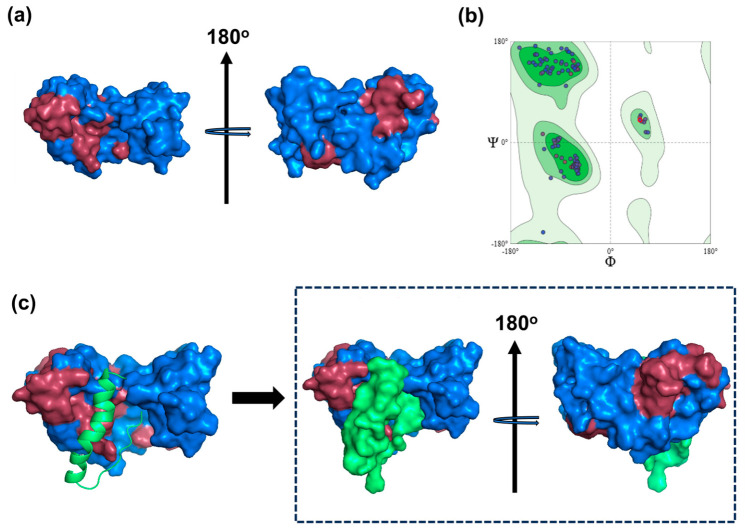
Modeled HPV-16 E6 protein: (**a**) E6 structure showing the conserved region highlighted in red; (**b**) Ramachandran plot showing the distribution of backbone dihedral angles (Φ and Ψ). The dark green contours indicate conformations free from steric clashes and therefore represent the most favorable regions. The lighter green contours correspond to additional permissible regions. In contrast, the white areas denote disallowed regions where steric interference occurs between side chain and backbone atoms; (**c**) molecular complex formation between E6 and p53 (green).

The HPV-16 E6 protein sequence was effectively modelled through homology modelling using SWISS-MODEL (Figure 2a). A protein model is deemed identical and valid when the sequence similarity to its template is approximately 20% [22]. In this study, the E6 HPV-16 model attained a similarity score of 85.42%. Conserved regions were identified in the model, as indicated in red in Figure 2a,c. Table 2 presents the sequence details of the E6 conserved region.

The quality of protein model was assessed using a Ramachandran plot, revealing that 97.83% of the residues are situated within favored regions (Figure 2b). A structure exhibiting more than 90% of residues in favored regions on the Ramachandran plot is deemed to possess high quality and accuracy. This finding indicates that most backbone dihedral angles (φ and ψ) adopt permissible, energetically favorable conformations typically observed in well-resolved secondary structures like α-helices and β-sheets [24].

After identifying the conserved region of HPV-16 E6, molecular docking analysis of its interaction with p53 revealed that the modeled protein could form an E6/p53 molecular complex, primarily engaging the conserved region (Figure 2c), with binding energies presented in Table 3. The top-ranked pose, involving 186 members, exhibited the lowest docking score of −1018.6 kcal/mol. More negative binding energy values indicate a more favorable interaction, and therefore, are used to rank E6–p53 complexes according to their predicted stability [25].

### 2.3. Analysis of Molecular Interaction and Stability P. urinaria with E6 HPV-16

Molecular docking simulations demonstrated that all compounds from *P. urinaria* can form molecular complexes with HPV-16 E6, involving interactions such as hydrogen bonds, van der Waals forces, and hydrophobic contacts (Table 4 and Table 5, and Supplementary Table S2). More negative binding affinity values indicate stronger and more stable interactions [26]. This study identified DG(17:1(9Z)/17:2(9Z,12Z)/0:0)[iso2] and 2,5-Bis[(2-acetamidobenzoyl)amino]-1,2,5,6-tetradeoxy-1,6-diphenyl-L-altritol as the compounds exhibiting the most negative binding affinity, demonstrating specific interaction with the conserved region of HPV-16 E6, as highlighted in bold in Table 5 and Supplementary Table S2. Interactions with the conserved region are essential for assessing ligand efficacy in inhibiting HPV-16 E6 function and can significantly influence the stability and activity of the viral protein [27].

**Figure 3:**
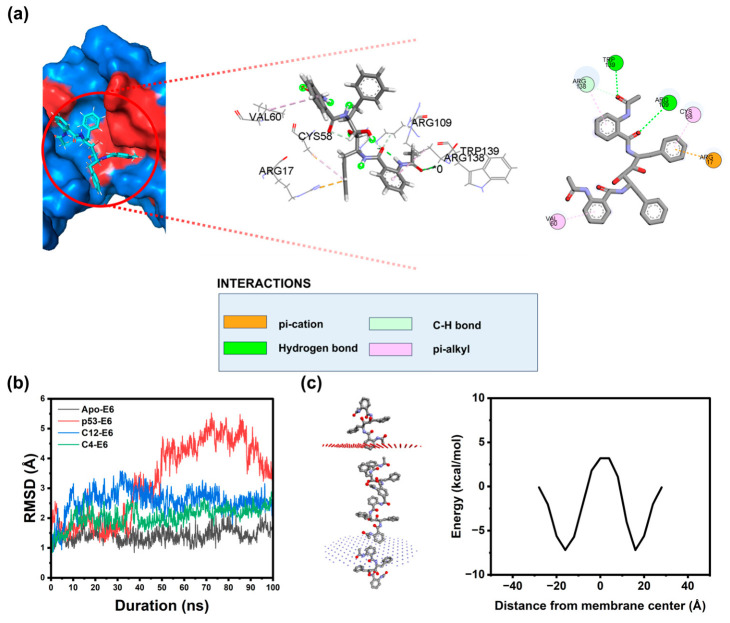
Interaction between ligands and E6: (**a**) molecular docking visualization of 2,5-Bis[(2-acetamidobenzoyl)amino]-1,2,5,6-tetradeoxy-1,6-diphenyl-L-altritol (*0) and E6, (**b**) alpha-carbon RMSD analysis after molecular dynamic simulation 100 ns, and (**c**) ability to penetrate the cell membrane.

Molecular dynamics simulations were conducted to assess ligand–protein stability, utilizing the alpha-carbon RMSD as an indicator of protein stability over a duration of 100 ns (Figure 3b). The two compounds with the highest binding affinity; the best results were obtained with 2,5-Bis[(2-acetamidobenzoyl)amino]-1,2,5,6-tetradeoxy-1,6-diphenyl-L-altritol with code C4 as the most stable, where during the simulation the average RMSD value of this compound was 2 Å, whereas E6-C12 (DG(17:1(9Z)/17:2(9Z,12Z)/0:0)[iso2]) reached > 2 Å, and the E6–p53 complex attained values as high as 5 Å. The results demonstrate that the ligand promotes a conformationally stable state in E6 relative to p53 binding [28].

### 2.4. Cell Membrane Permeability Computational Evaluation

Figure 3c shows the membrane permeability profile of 2,5-Bis[(2-acetamidobenzoyl)amino]-1,2,5,6-tetradeoxy-1,6-diphenyl-L-altritol across the lipid bilayer. As the compound approaches the bilayer center (around 0 Å), the free energy increases, indicating the presence of a significant thermodynamic barrier to full transbilayer passage. This pattern implies that passive uptake is not maximal and that intracellular delivery to the E6 protein target could be improved by optimized delivery strategies, such as nanodrug formulation [29].

### 2.5. Anticancer Activity of P. urinaria

**Figure 4:**
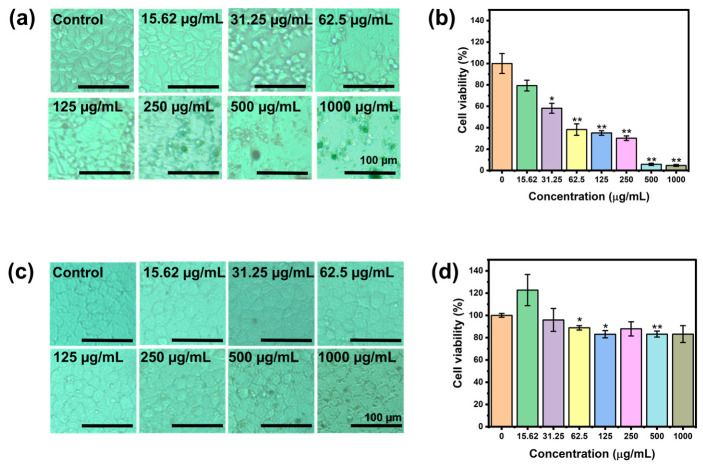
Cell morphology (**a**,**c**) and cytotoxicity assays (**b**,**d**) following *P. urinaria* treatment in HeLa (**top**) and HaCaT (**bottom**) cells. Statistical significance: *p* < 0.05 (*) and *p* < 0.01 (**) based on *t*-test analysis.

**Figure 5:**
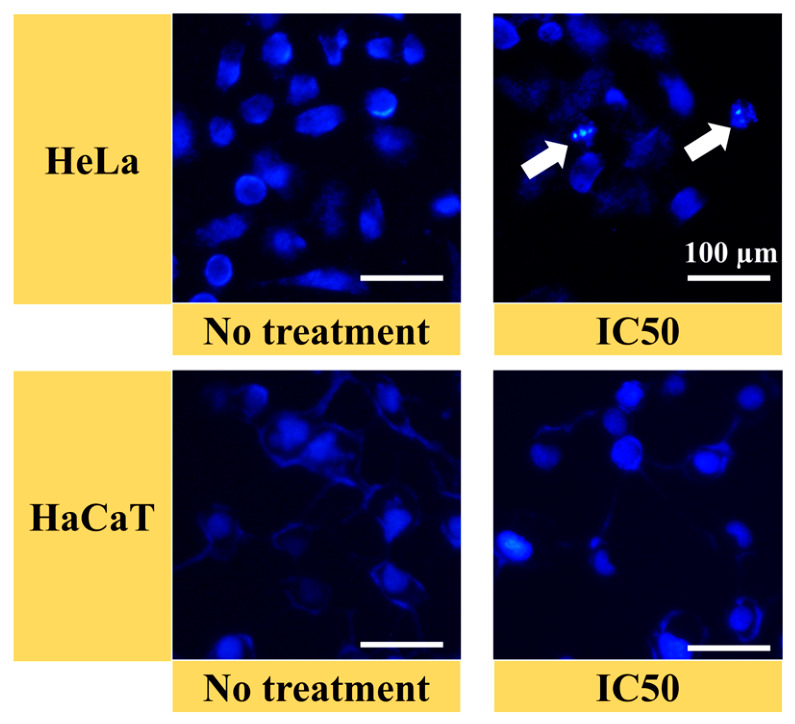
Apoptotic cells after treatment (white arrow indicate apoptotic cell).

This study evaluated the cytotoxic effects of *P. urinaria* compounds on cancer cells. Figure 4a,b illustrate that the MTT assay indicated a significant reduction in cervical cancer cell viability by *P. urinaria* within 24 h, starting at a concentration of 31.25 µg/mL (*p* < 0.05), with an IC_50_ value determined to be 49.26 µg/mL (Figure S5a). An IC_50_ value ranging from 20 to 100 µg/mL is categorized as demonstrating moderate cytotoxic activity according to established criteria [30]. Furthermore, morphological changes consistent with apoptosis were observed in treated cancer cells, as illustrated in Figure 5.

In contrast, testing on HaCaT cells yielded different results, generally showing no marked dose-dependent decrease in HaCaT cell viability across concentrations up to 1000 μg/mL (Figure 4c,d), with an IC_50_ value of 2421.77 μg/mL (Supplementary Figure S5b) and a resulting selectivity index (SI) of 47.31. An SI value greater than 3 is categorized as highly selective [31]. However, it is important to note that HaCaT cells are human keratinocytes characterized by high keratin content and specialized membrane structures, providing a robust barrier function that makes it difficult for cytotoxic compounds to penetrate the cells [32]. This property likely contributes to increased resistance to the extract and may partly reflect differences in cellular uptake or permeability rather than solely intrinsic drug selectivity for cancer cells over normal cells.

## 3. Discussion

This study effectively modeled the HPV-16 E6 protein, which, although its sequence is accessible in databases, lacks a fully resolved structural model to date [33]. E6 plays a crucial role in viral infection and carcinogenesis. Prior antiviral studies, including those involving retroviral agents like tipranavir, have employed a comparable strategy that focuses on conserved regions to impede multidrug-resistant HIV protease [34]. Identifying conserved regions is essential, as these segments remain unchanged across different strains. Consequently, drugs aimed at these regions can inhibit viral activity by attaching to sites that are less prone to mutation, thereby enhancing their efficacy across a wider array of HPV-16 strains [35].

The E6 protein promotes p53 degradation by forming a complex with E6-associated protein (E6AP), leading to p53 ubiquitination and proteasomal degradation. Consequently, infected cells exhibit a loss of cell cycle regulation and undergo uncontrolled proliferation [36]. Compounds from *P. urinaria* exhibited more stable binding to E6 compared to p53, highlighting a potential therapeutic intervention pathway. Inhibition of E6 restores p53 function, facilitating DNA repair, cell cycle regulation, and the selective induction of apoptosis to diminish or eradicate HPV-infected cells [4]. Moreover, E6 disrupts immune detection of the virus by impairing interferon signaling pathways, including TYK2 and IRF3. Therefore, inhibiting E6 may improve immune recognition and clearance of HPV, which further potentially enhance the probability of patients attaining undetectable HPV levels [37]. Computational screening revealed that 2,5-Bis[(2-acetamidobenzoyl)amino]-1,2,5,6-tetradeoxy-1,6-diphenyl-L-altritol exhibited the most significant binding affinity for the E6 oncoprotein. Previous study mentioned that the molecule belongs to diol class of human immunodeficiency virus type 1 (HIV-1) inhibitor. 2,5-Bis[(2-acetamidobenzoyl)amino]-1,2,5,6-tetradeoxy-1,6-diphenyl-L-altritol could bind symmetrically with the central hydroxyl positioned between the two catalytic Asp25 residues of HIV-1 protease [38].

The MTT assay demonstrated marked cytotoxic effects of *P. urinaria*-derived compounds on cervical cancer cells, plausibly mediated by well-characterized constituents, including d-δ-tocopherol (a potent inducer of apoptosis), ursolic acid (antiproliferative and pro-apoptotic), ergosta-type sterols (apoptosis-inducing and antiproliferative), and pheophorbide A (antiproliferative) [39,40,41,42]. The compounds contain diacylglycerol derivatives that are able to exert anticancer actions via the Ras/ERK signaling pathway, paralleling the mechanism of bryostatin-1, a patented diacyglycerol mimetic currently under clinical investigation [43]. Collectively, these data suggest that *P. urinaria*-derived compounds represent a multimodal phytochemical platform with promising potential as a therapeutic or adjuvant strategy for cervical cancer.

## 4. Materials and Methods

### 4.1. Plant Identification, Extraction, and Metabolite Annotation

Plant specimens were gathered in Tasikmalaya, West Java, Indonesia. The samples were later conveyed to the Plant Systematics Laboratory of Universitas Gadjah Mada for identification. The findings validated that the gathered specimens were *Phyllanthus urinaria* L. (Reference letter number: 00882/S.Tb./IV/2025).

The extraction procedure was modified from the maceration technique outlined by Naviglio et al. (2023) [44], employing a ratio of 1:10 for plant material to absolute ethanol (Merck, Darmstadt, Germany). The mixture was allowed to rest for 72 h with periodic agitation and subsequently filtered using filter paper having a pore width of 15–20 µm. The filtrate was evaporated with a rotary evaporator (Eyela, Tokyo, Japan) to get a paste-like extract [44]. Fractionation was conducted by dissolving 1 g of extract in 10 mL of 20% ethanol, combining it with 10 mL of n-hexane (Merck, Germany), and permitting the mixture to rest for 30 min in a separatory funnel. The ethanol and n-hexane phases were isolated, and this procedure was conducted three times. The fractionated product was subsequently evaporated to produce a dense, sticky paste [45].

Metabolite annotation was performed with Liquid Chromatography–High Resolution Mass Spectrometry (LC-HRMS). An aliquot of 1 mg of extract was diluted in 1 mL of ethyl acetate and analyzed via a Thermo Scientific™ Vanquish™ Horizon UHPLC system equipped with a Binary Pump (Germering, Germany) and a Thermo Scientific™ Orbitrap™ Exploris 240 HRMS (Bremen, Germany). The samples were injected and analyzed under controlled conditions: chromatographic separation was conducted on a reversed-phase C18 column (2.1 × 100 mm, 1.9 µm) at a flow rate of 0.3 mL/min and 40 °C, employing a 5–95% gradient of solvent B (0.1% formic acid). Mass spectrometric data were acquired in both full scan mode (resolution: 120,000 FWHM, *m*/*z* range: 40–800) and dd-MS^2^ mode (~30,000 FWHM) with normalized collision energy (NCE) ranging from 20 to 40. EASY-IC internal standards were utilized to uphold calibration, guaranteeing mass accuracy beneath 1 ppm. An AGC target of roughly 1 × 10^6^ to 1 × 10^7^ was employed, utilizing automated injection time to optimize signal strength. Data processing and compound annotation were conducted using Thermo Scientific™ Compound Discoverer 3.3 (San Jose, CA, USA). Adducts [M + H]^+^ and [M − H]^–^ were analyzed. The LC-HRMS data consisted of MS1 ion spectra (*m*/*z*) containing the exact mass, isotopic pattern, and reference ions, which were subsequently compared with a spectral library (mzCloud) and a structure database (ChemSpider). Candidate compounds detected in MS1 were required to have a delta mass annotation of ≤5 ppm. To confirm that the proposed chemical formula was structurally plausible, the corresponding MS2 spectra were examined to identify characteristic fragment ions supporting the putative structure.

Compound annotation followed a hierarchical matching strategy. Features with associated MS1 spectra were first searched against the mzCloud spectral library. Library-based annotations were validated based on agreement of diagnostic fragment ions, acceptable mass deviation, and isotopic pattern consistency; compounds annotated through this approach were assigned Level 2 (probable structure) confidence, with a match score > 80% required to be classified as a good match. In the absence of a corresponding reference spectrum in mzCloud, features were annotated using formula-based databases, including ChemSpider, by accurate mass and molecular formula matching, and compounds identified solely through this approach were categorized as Level 3 (tentative candidates) [46,47,48].

The LC-HRMS analysis was performed in a semi-quantitative manner, in which the peak area in the extracted ion chromatogram (EIC) was used as a surrogate for the relative abundance of each compound, and only compounds contributing more than 1% of the total peak area of all detected features were selected for further analysis to focus on components with quantitatively significant contributions.

### 4.2. Homology Modelling and Structure Assessment of E6 HPV-16 Protein

The E6 HPV-16 sequence was retrieved from the NCBI database (https://www.ncbi.nlm.nih.gov/). Protein modelling was conducted utilising SWISS-MODEL (https://swissmodel.expasy.org/) with a suitable E6 HPV-16 template, followed by structural evaluation to assess the quality, physicochemical, and stereochemical attributes of the modelled protein to ensure its reliability for subsequent biological analysis [49]. Conserved areas within the protein were discovered, as previously described by Kharisma et al. (2020), utilising data from 400 HPV-16 strains [23].

### 4.3. Molecular Interaction Analysis of E6–p53 and E6-Compounds via Molecular Docking

Molecular interactions between the E6 protein and p53 were examined by blind molecular docking utilizing ClusPro 2.0 (https://cluspro.org/), which effectively facilitates precise predictions of protein–protein binding modes [50]. The interaction between E6 and the isolated *P. urinaria* compound was examined utilizing Molecular Operating Environment (MOE) version 2022.02. Ligand preparation involved energy minimization utilizing the MMFF94 force field with a root mean square (RMS) gradient threshold of 0.001 kcal/mol/Å, whereas the E6 protein was pre-processed employing the AMBER10:EHT force field with the identical RMS gradient. Binding site prediction was performed using MOE’s “site finder” tool, with dummy ligand atoms strategically placed as binding sites throughout the docking process [51]. Molecular docking utilized the induced fit methodology, employing the Triangle Matcher/London dG approach for ligand placement and the Forcefield/GBVI-WSA dG for refinement. The molecular docking experiment produced 30 poses, from which the five highest-ranked conformations were chosen for additional study [52].

### 4.4. Molecular Dynamic Simulation

Molecular dynamics simulations were conducted using YASARA Dynamics 21.6.7 version (Biosciences, Vienna, Austria) to validate molecular docking results and assess the stability and behavior of the protein in settings closely mimicking the physiological cellular environment. The simulation was performed at 312 K and pH 7.4 for 100 ns, employing the AMBER03 force field. Following the simulation, the ‘md_analyze’ module was employed to provide the Root Mean Square Deviation (RMSD) data [53].

### 4.5. Analysis of Membrane Permeability

The capacity of substances to traverse the cell membrane was evaluated utilizing the PerMM web tool (https://permm.phar.umich.edu/). The environmental parameters were established to replicate healthy cellular conditions, especially a pH of 7.4 and a temperature of 310 K.

### 4.6. In Vitro Anticancer Activity of P. urinaria

Human epidermal keratinocyte (HaCaT) cells and human cervical adenocarcinoma (HeLa) cells were obtained from Faculty of Medicine, Universitas Indonesia, and cultivated in their designated complete media. HaCaT cells were grown in Dulbecco’s Modified Eagle Medium (DMEM, Sigma-Aldrich, Burlington, MA, USA) supplemented with 10% fetal bovine serum (FBS, Sigma-Aldrich, USA) and 1% penicillin-streptomycin (Sigma-Aldrich, MA, USA). HeLa cells were cultured in Minimum Essential Medium (MEM, Servicebio, Hubei, China) augmented with 10% fetal bovine serum and 1% penicillin-streptomycin. Upon achieving confluence, cells were inoculated into 96-well plates at a density of 10,000 cells per well and incubated for 24 h. The wells were subsequently treated with *P. urinaria* extract at doses of 15.62, 31.25, 62.5, 125, 250, 500, and 1000 µg/mL for a duration of 24 h. Cell viability in each well was later evaluated quantitatively using the 3-[4,5-dimethylthiazol-2-yl]-2,5-diphenyl tetrazolium bromide (MTT, Servicebio, China) assay. In addition, apoptotic activity of the cells after treatment employed DAPI staining (ABP Bio, Rockville, MD, USA) after that were observed using a Zeiss Axio Vert.A1 FL inverted microscope (Carl Zeiss Microscopy GmbH, Jena, Germany).

### 4.7. Statistical Analysis

The results of the in-silico analysis are displayed through figures and tables, and subsequently, were evaluated against standards. In vitro data regarding cell viability are presented as mean ± standard error (SE) and analyzed using linear regression to determine the IC_50_ values. Furthermore, the cell viability at each concentration was subjected to statistical comparison with the control group through the application of *t*-tests. Upon acquiring the IC_50_ values, the selectivity index of *P. urinaria* was determined through the application of the subsequent Equation (1):

Selectivity Index (SI) = IC_50_ normal cells/IC_50_ cancer cells (1)

## 5. Conclusions

This study suggests that bioactive constituents present in *P. urinaria* are predicted, based on molecular docking, to bind to the HPV-16 E6 oncoprotein and thereby potentially interfere with its interaction with p53. Among the evaluated compounds, 2,5-Bis[(2-acetamidobenzoyl)amino]-1,2,5,6-tetradeoxy-1,6-diphenyl-L-altritol emerged from molecular dynamics simulations as a particularly promising candidate for this mechanism. In vitro assays demonstrated that the *P. urinaria* n-hexane fraction reduced cervical cancer cell viability (IC_50_ = 49.26 µg/mL) while exhibiting a high selectivity index toward normal cells (SI = 47.31). Nevertheless, the predicted ability of compounds to inhibit E6 remains an in silico and should be validated in future studies using specific in vitro approaches. Overall, the integrated in silico and in vitro findings support *P. urinaria* as a promising source of bioactive molecules for further development as potential therapeutics for HPV-associated cervical cancer.

## Figures and Tables

**Table 1:** Major compounds in non-polar fraction of *P. urinaria* leaves.

ID	Compound	IUPAC Name	Chemical Formula	Chemical Groups	Identification Confidence
1	DG(18:3(9Z,12Z,15Z)/18:3(9Z,12Z,15Z)/0:0)	[(2*S*)-3-hydroxy-2-[(9*Z*,12*Z*,15*Z*)-octadeca-9,12,15-trienoyl]oxypropyl] (9*Z*,12*Z*,15*Z*)-octadeca-9,12,15-trienoate	C_39_H_64_O_5_	Glycerolipids	Level 3
2	Ceriporic acid C	2-[(*Z*)-hexadec-7-enyl]-3-methylidenebutanedioic acid	C_21_H_36_O_4_	Fatty Acyls	Level 3
3	6-[1]-ladderane hexanol	6-(1,2,4a,4b,5,6,8a,8b-octahydrobiphenylen-2-yl)hexan-1-ol	C_18_H_28_O	Fatty Acyls	Level 3
4	2,5-Bis[(2-acetamidobenzoyl)amino]-1,2,5,6-tetradeoxy-1,6-diphenyl-L-altritol	2-acetamido-N-[(2S,3S,4R,5S)-5-[(2-acetamidobenzoyl)amino]-3,4-dihydroxy-1,6-diphenylhexan-2-yl]benzamide	C_36_H_38_N_4_O_6_	Peptidomimetics	Level 3
5	Echinenone	2,4,4-trimethyl-3-[(1E,3E,5E,7E,9E,11E,13E,15E,17E)-3,7,12,16-tetramethyl-18-(2,6,6-trimethylcyclohexen-1-yl)octadeca-1,3,5,7,9,11,13,15,17-nonaenyl]cyclohex-2-en-1-one	C_40_H_54_O	Prenol lipids	Level 3
6	Isoheptadecasphinganine	(2S,3R)-2-amino-15-methylhexadecane-1,3-diol	C_17_H_37_NO_2_	Sphingolipid	Level 3
7	3β-Hydroxy-4β-methyl-5α-cholest-7-ene-4α-carboxylate	(3S,4S,5R,9R,10R,13R,14R,17R)-3-hydroxy-4,10,13-trimethyl-17-[(2R)-6-methylheptan-2-yl]-1,2,3,5,6,9,11,12,14,15,16,17-dodecahydrocyclopenta[a]phenanthrene-4-carboxylate	C_29_H_48_O_3_	Steroid acid anion	Level 3
8	Pheophorbide A	3-[(3R,21S,22S)-16-ethenyl-11-ethyl-4-hydroxy-3-methoxycarbonyl-12,17,21,26-tetramethyl-7,23,24,25-tetrazahexacyclo [18.2.1.15,8.110,13.115,18.02,6]hexacosa-1,4,6,8(26),9,11,13(25),14,16,18(24),19-undecaen-22-yl]propanoic acid	C_35_H_36_N_4_O_5_	Pheophorbide	Level 3
9	1-Palmitoylglycerophosphocholine	2-[(3-hexadecanoyloxy-2-hydroxypropoxy)-hydroxyphosphoryl]oxyethyl-trimethylazanium	C_24_H_50_NO_7_P	Glycerophospholipid	Level 3
10	Certonardosterol J	(3S,5S,6S,8R,9S,10R,13R,14S,15R,17R)-17-[(E,2R,5R,6R)-7-hydroxy-4,5,6-trimethylhept-3-en-2-yl]-10,13-dimethyl-2,3,4,5,6,7,8,9,11,12,14,15,16,17-tetradecahydro-1H-cyclopenta[a]phenanthrene-3,6,15-triol	C_29_H_50_O_4_	Sterol lipids	Level 3
11	Dracontioside A	(2R)-2-hydroxy-N-[(2S,3R,4E,8Z)-3-hydroxy-1-[(2R,3R,4S,5S,6R)-3,4,5-trihydroxy-6-(hydroxymethyl)oxan-2-yl]oxyoctadeca-4,8-dien-2-yl]octadecanamide	C_42_H_79_NO_9_	Sphingolipids	Level 3
12	DG(17:1(9Z)/17:2(9Z,12Z)/0:0)[iso2]	[(2S)-2-[(9Z,12Z)-heptadeca-9,12-dienoyl]oxy-3-hydroxypropyl] (Z)-heptadec-9-enoate	C_37_H_66_O_5_	Glycerolipids	Level 3
13	Ergosta-3,5-diene	(8S,9S,10R,13R,14S,17R)-17-[(2R)-5,6-dimethylheptan-2-yl]-10,13-dimethyl-2,7,8,9,11,12,14,15,16,17-decahydro-1H-cyclopenta[a]phenanthrene	C_28_H_46_	Sterol	Level 3
14	2-Amino-1,3,4-octadecanetriol	2-amino-1,3,4-octadecanetriol	C_18_H_39_NO_3_	Sphingoid	Level 2
15	PC(18:3(9Z,12Z,15Z)/0:0)	[(2R)-2-hydroxy-3-[(9Z,12Z,15Z)-octadeca-9,12,15-trienoyl]oxypropyl] 2-(trimethylazaniumyl)ethyl phosphate	C_26_H_48_NO_7_P	Glycerophospholipids	Level 3
16	Ursolic acid	(1S,2R,4aS,6aR,6aS,6bR,8aR,10S,12aR,14bS)-10-hydroxy-1,2,6a,6b,9,9,12a-heptamethyl-2,3,4,5,6,6a,7,8,8a,10,11,12,13,14b-tetradecahydro-1H-picene-4a-carboxylic acid	C_30_H_48_O_3_	Prenol lipids	Level 2
17	11-oxo-undeca-5,8-dienoic acid	(5Z,8Z)-11-oxoundeca-5,8-dienoic acid	C_11_H_16_O_3_	Fatty acyls	Level 3
18	PA(18:2(9Z,12Z)/0:0)	[(2R)-2-hydroxy-3-phosphonooxypropyl] (9Z,12Z)-octadeca-9,12-dienoate	C_21_H_39_O_7_P	Glycerophospholipids	Level 3
19	4,4-dimethyl-5a-cholesta-8,24-dien-3-b-ol	(3S,5R,10S,13R,14R)-4,4,10,13-tetramethyl-17-[(2R)-6-methylhept-5-en-2-yl]-1,2,3,5,6,7,11,12,14,15,16,17-dodecahydrocyclopenta[a]phenanthren-3-ol	C_29_H_48_O	Prenol lipids	Level 3
20	Myristyl sulfate	Tetradecyl hydrogen sulfate	C_14_H_30_O_4_S	Alkyl sulfate	Level 3
21	D-δ-tocopherol	(2R)-2,8-dimethyl-2-[(4R,8R)-4,8,12-trimethyltridecyl]-3,4-dihydrochromen-6-ol	C_27_H_46_O_2_	Prenol Lipids	Level 2
22	2-Dodecylbenzenesulfonic acid	2-dodecylbenzenesulfonic acid	C_18_H_30_O_3_S	Benzenoid	Level 3
23	4-Undecylbenzenesulfonic acid	4-undecylbenzenesulfonic acid	C_17_H_28_O_3_S	Benzenoid	Level 3

**Table 2:** Conserved region of E6 HPV-16 [23].

Sequence Position	Amino Acid Sequences	Length (mer)
37–41	CVYCK	5
91–101	YGTTLEQQYNK	11
108–115	IRCINCQK	7
121–127	EKQRHLDK	8
136–149	RGRWTGRCMSCCRS	14

**Table 3:** Molecular interaction between E6 and p53.

Cluster	Members	Docking Score (kcal/mol)
0	186	−1018.6
1	111	−1037.0
2	108	−1057.1
3	99	−967.6
4	62	−953.2
5	59	−1033.0

**Table 4:** Molecular interaction *P. urinaria* and E6 HPV-16.

Compound	Binding Affinity (kcal/mol)	RMSD (Å)
Ergosta-3,5-diene	−6.65	1.61
4,4-dimethyl-5a-cholesta-8,24-dien-3-b-ol	−6.72	1.79
1-Palmitoylglycerophosphocholine	−7.75	1.67
2,5-Bis[(2-acetamidobenzoyl)amino]-1,2,5,6-tetradeoxy-1,6-diphenyl-L-altritol	−8.16	1.73
2-Amino-1,3,4-octadecanetriol	−6.64	1.08
2-Dodecylbenzenesulfonic acid	−6.35	1.97
3β-Hydroxy-4β-methyl-5α-cholest-7-ene-4α-carboxylate	−6.54	1.22
4-Undecylbenzenesulfonic acid	−6.28	1.28
6-[1]-ladderane hexanol	−6.06	1.20
11-oxo-undeca-5,8-dienoic acid	−5.29	1.65
Ceriporic acid C	−7.07	1.89
Certonardosterol J	−6.96	1.32
D-δ-Tocopherol	−7.75	1.95
DG(18:3(9Z,12Z,15Z)/18:3(9Z,12Z,15Z)/0:0)	−8.05	1.79
DG(17:1(9Z)/17:2(9Z,12Z)/0:0)[iso2]	−8.93	1.53
Dracontioside A	−8.81	1.93
Echinenone	−6.96	1.53
Isoheptadecasphinganine	−6.76	1.87
Myristyl sulfate	−6.44	1.66
PA(18:2(9Z,12Z)/0:0)	−7.03	0.91
PC(18:3(9Z,12Z,15Z)/0:0)	−7.82	1.56
Pheophorbide A	−7.19	1.60
Ursolic acid	−6.50	1.37

**Table 5:** Interactions of best compound with E6 HPV-16.

Compound	Interaction			
	Hydrogen	Carbon Hydrogen	van der Waals	Hydrophobic
DG(17:1(9Z)/17:2(9Z,12Z)/0:0)[iso2]	Cys58	-	Val69, Leu74, Leu57, **Gln114**, Phe52, **Arg136**, Ser81, Arg84, Arg62, Val60, **Val38**, **Gly137**, **Lys101**, Asp105	**Tyr39**, **Arg109**, **Arg138**, Leu106, **Tyr99**, **Trp139**, Leu107, His85, Tyr77, Ile80
2,5-Bis[(2-acetamidobenzoyl)amino]-1,2,5,6-tetradeoxy-1,6-diphenyl-L-altritol	**Trp139**, **Arg109**	**Arg138**	Cys58, Val60	Arg17

## Data Availability

The data that support the findings of this study are available from the corresponding author upon reasonable request.

## Supplementary Materials

The following supporting information can be downloaded at: https://www.mdpi.com/article/10.3390/ijms27114780/s1.

## Abbreviations

The following abbreviations are used in this manuscript:

AIR	Apoptosis-inducing factor
AGC	Automatic gain control
DMEM	Dulbecco’s modified eagle medium
dd-MS^s^	Data-dependent tandem mass spectrometry
E6AP	E6-associated protein
FBS	Fetal bovine serum
FWHM	Full width at half maximum
HDI	Human development index
IRF3	Interferon regulatory factor 3
LC-HRMS	Liquid chromatography-High resolution mass spectrometry
MEM	Minimum essential medium
MMFF94	Merck molecular force field 94
MOE	Molecular operating environment
MS	Mass spectrometry
MTT	3-(4,5-Dimethylthiazol-2-yl)-2,5-Diphenyltetrazolium Bromide
NCE	Normalized collision energy
NCBI	National center for biotechnology information
ORF	Open reading frame
p53	Tumor protein p53
RMSD	Root mean square deviation
ROS	Reactive oxygen species
SI	Selectivity index
SWISS-MODEL	Swiss Institute of Bioinformatic Modelling Server
TYK2	Tyrosine Kinase 2
