# Supplementary material for: Computational and Cytotoxicity Evaluation of Phyllanthus Urinaria-Derived Compounds as Potential Anti-Cervical Cancer Agents via HPV-16 E6 Oncoprotein Inhibition
Source: Int J Mol Sci. 2026 May 26;27(11):4780. doi: 10.3390/ijms27114780 (PMC13257223; doi:10.3390/ijms27114780)
Supplement: Supplementary file 1 [file ijms-27-04780-s001.zip › ijms-4138938-supplementary.pdf]

Supplementary Material

Supplementary Figure S1 Total ion chromatogram (TIC) of n-hexane fraction of *P. urinaria* leaves extract.

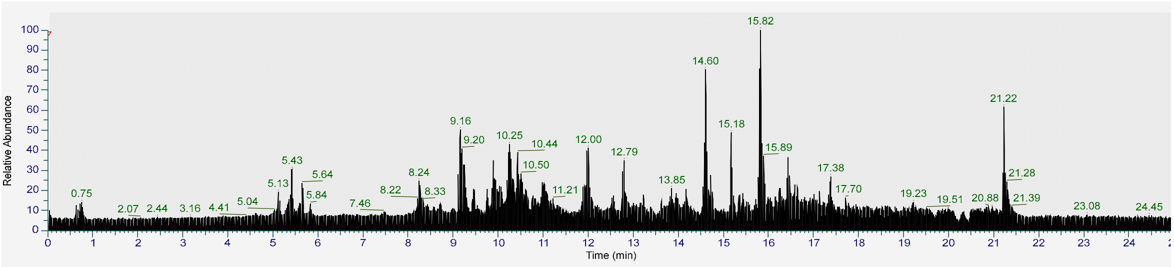

Supplementary Figure S2. Extracted ion chromatogram (EIC) of compounds

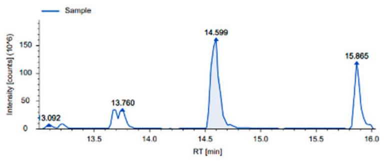

1

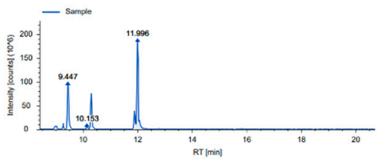

2

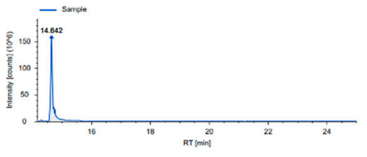

3

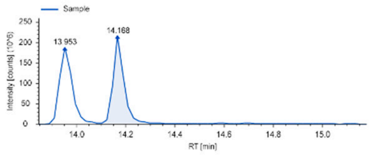

4

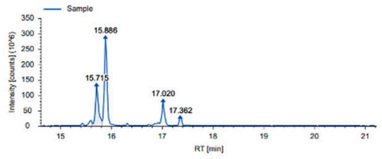

5

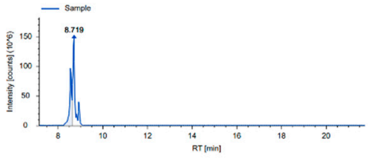

6

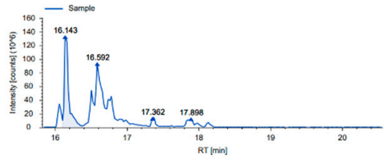

7

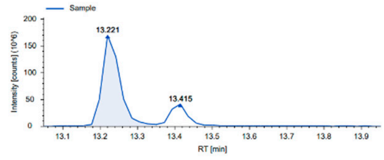

8

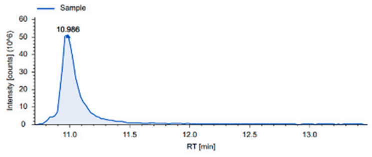

9

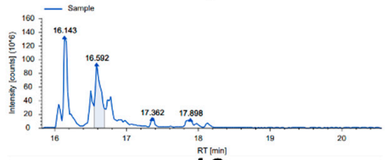

10

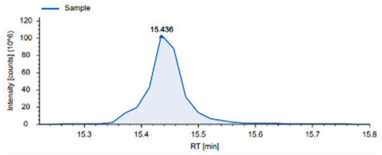

11

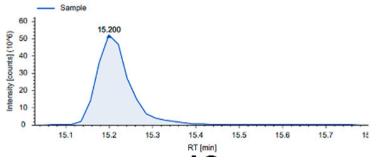

12

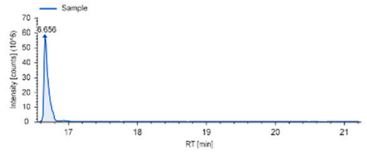

13

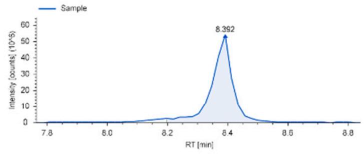

14

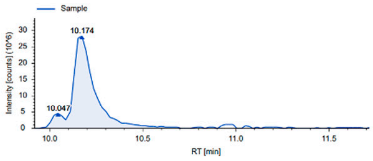

15

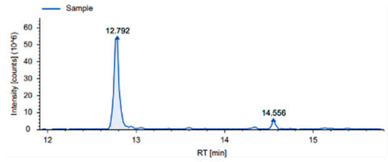

16

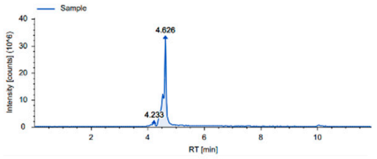

17

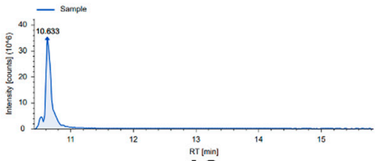

18

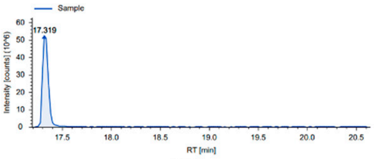
Sample
17.319
Intensity [counts] (10^6)
RT [min]

19

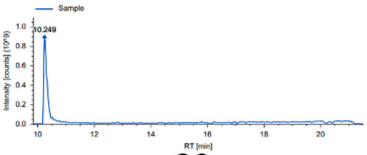
Sample
10.249
Intensity [counts] (10^9)
RT [min]

20

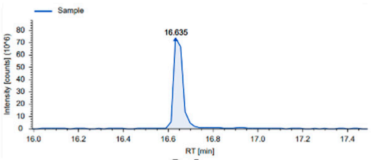
Sample
16.635
Intensity [counts] (10^6)
RT [min]

21

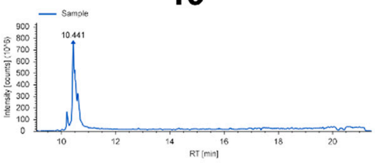
Sample
10.441
Intensity [counts] (10^6)
RT [min]

22

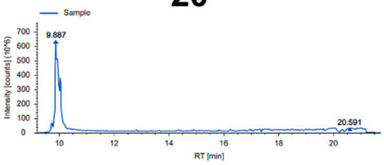
Sample
9.887
20.591
Intensity [counts] (10^6)
RT [min]

23

Supplementary Figure S3. MS^1 spectra of compound by LC-HRMS analysis

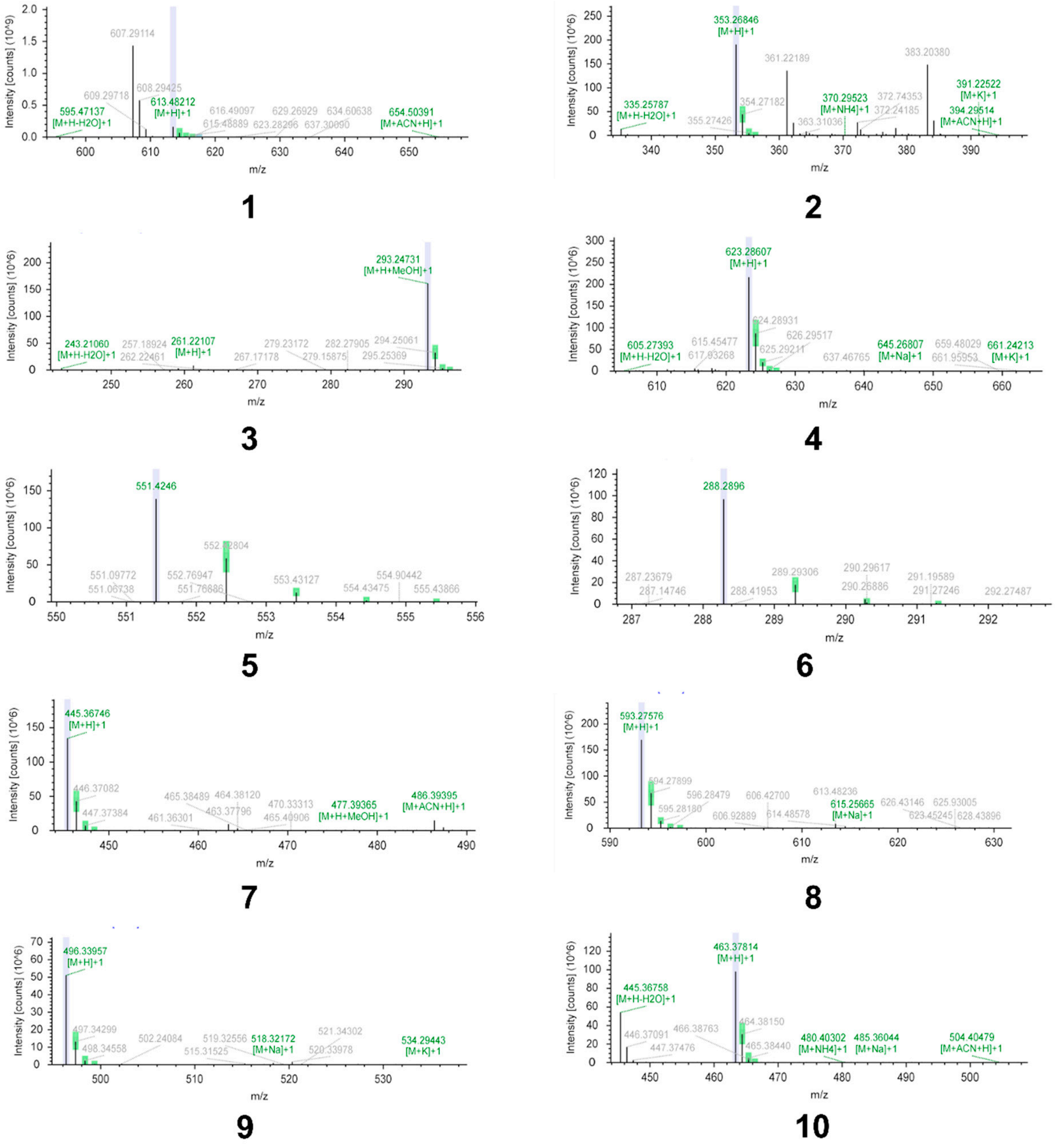

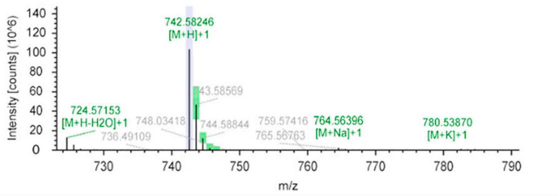

11

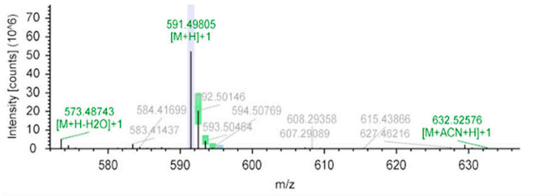

12

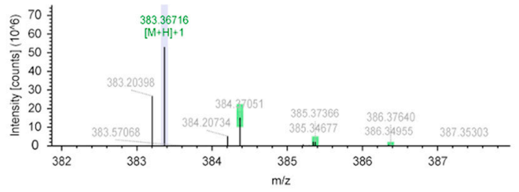

13

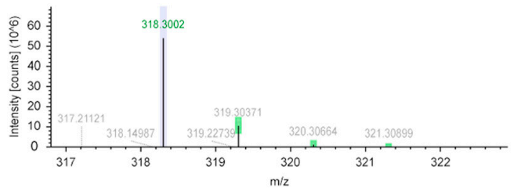

14

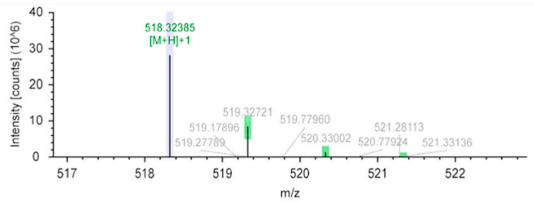

15

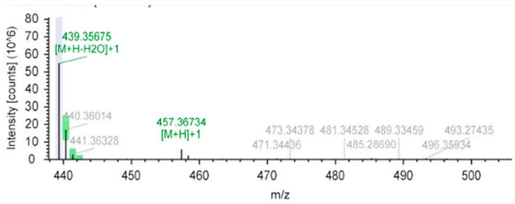

16

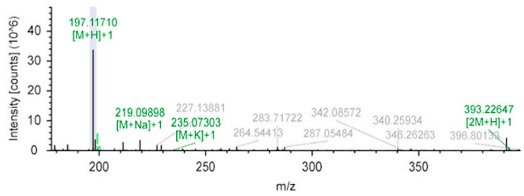

17

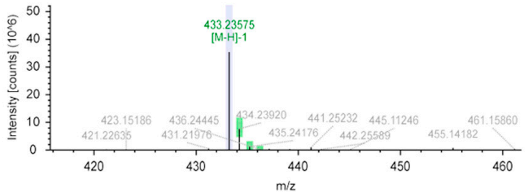

18

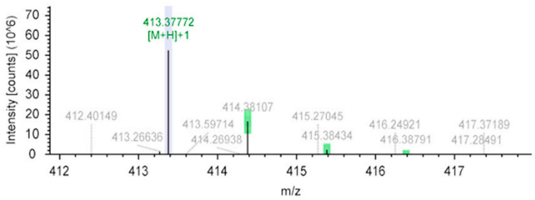

19

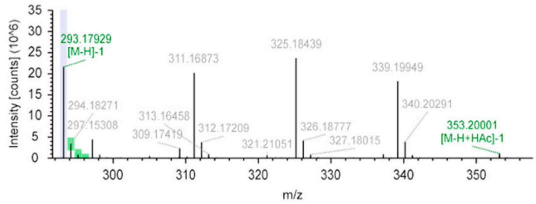

20

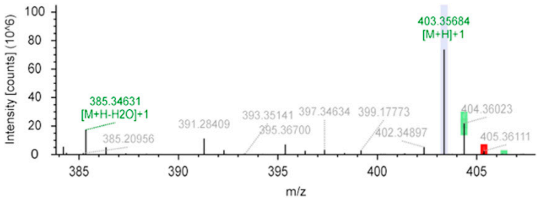
403.35684
[M+H]+1
385.34631
[M+H-H2O]+1
Intensity [counts] (10^6)
m/z

21

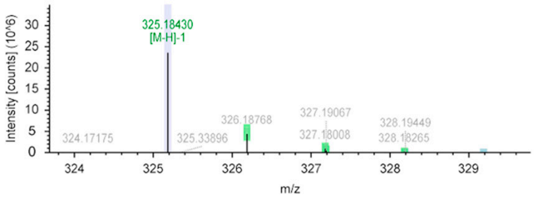
325.18430
[M-H]-1
Intensity [counts] (10^6)
m/z

22

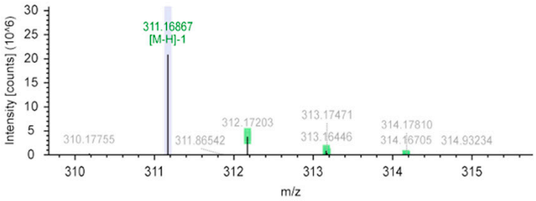
311.16867
[M-H]-1
Intensity [counts] (10^6)
m/z

23

Supplementary Figure S4. dd-MS² spectra and ion fragmentation analysis.

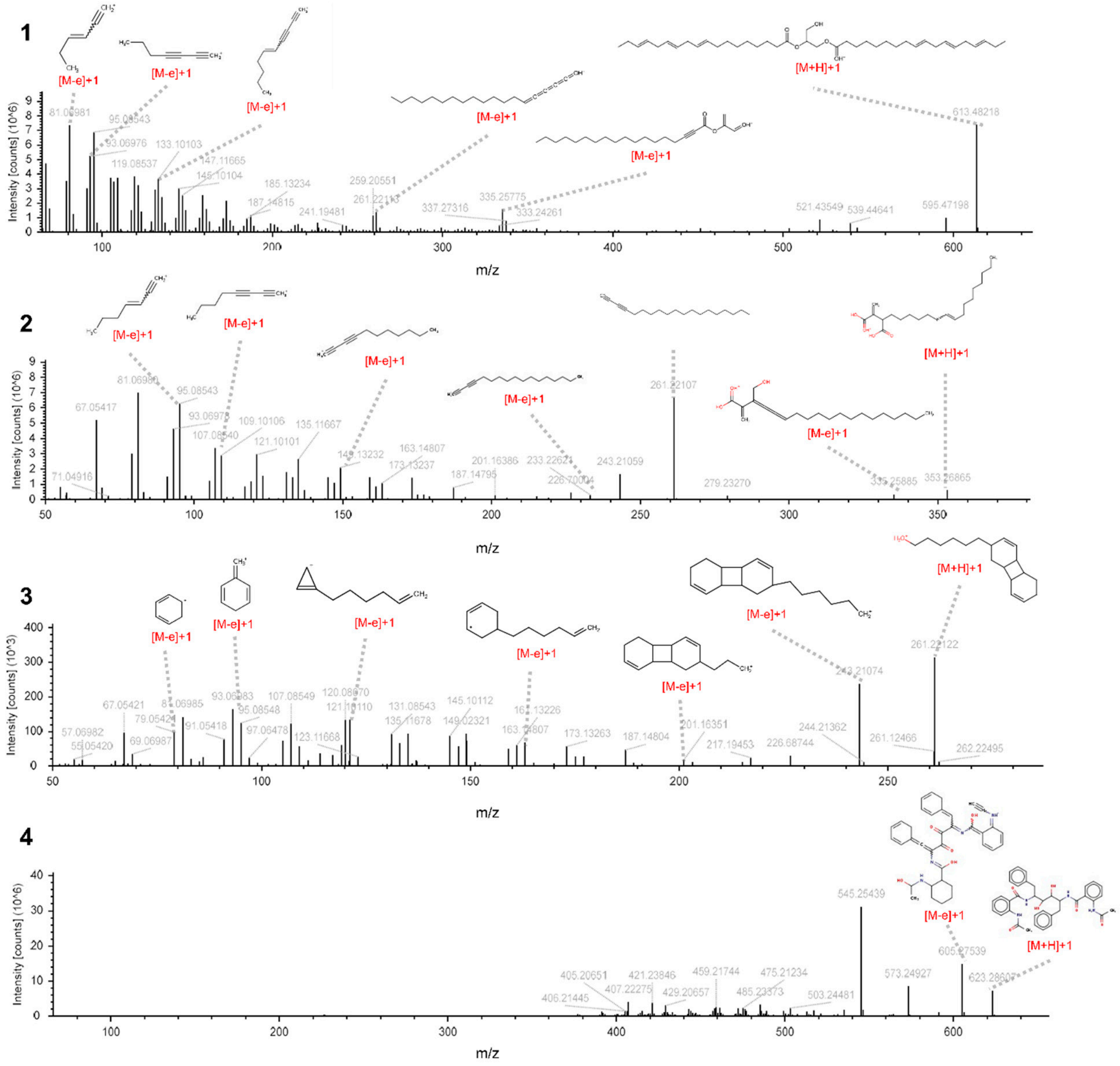

5

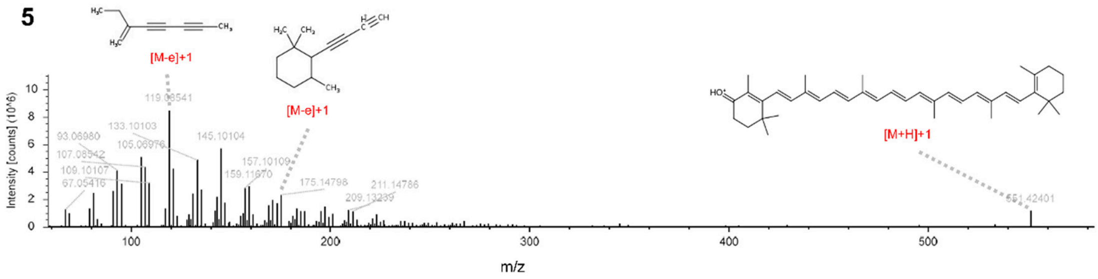
[M-e]+1
[M-e]+1
[M+H]+1
Intensity [counts] (10^6)
m/z
119.06541
133.10103
145.10104
157.10109
175.14798
211.14786
209.13239

6

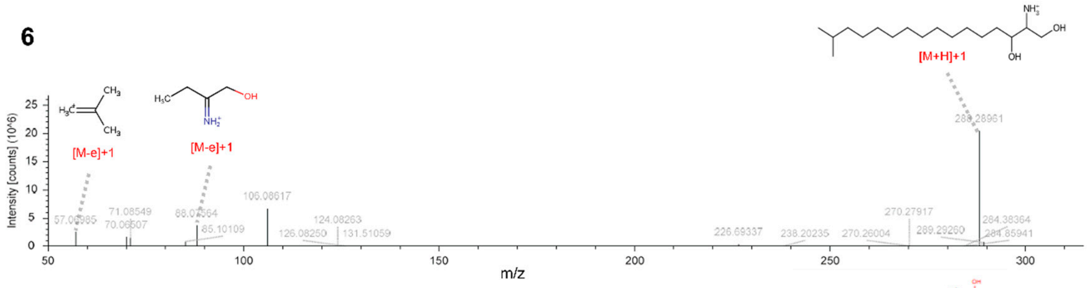
[M-e]+1
[M-e]+1
[M+H]+1
Intensity [counts] (10^6)
m/z
106.08617
124.08263
270.27917
284.38364

7

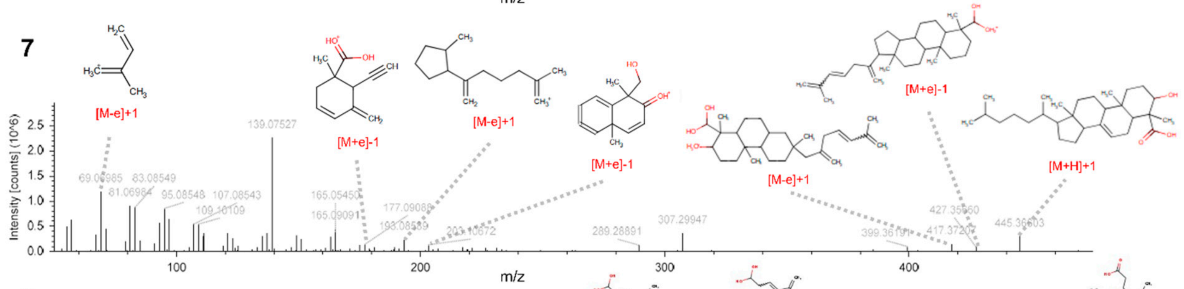
[M-e]+1
[M+e]-1
[M-e]+1
[M+e]-1
[M-e]+1
[M+e]-1
[M+H]+1
Intensity [counts] (10^6)
m/z
139.07527
307.29947
289.28891
445.36603

8

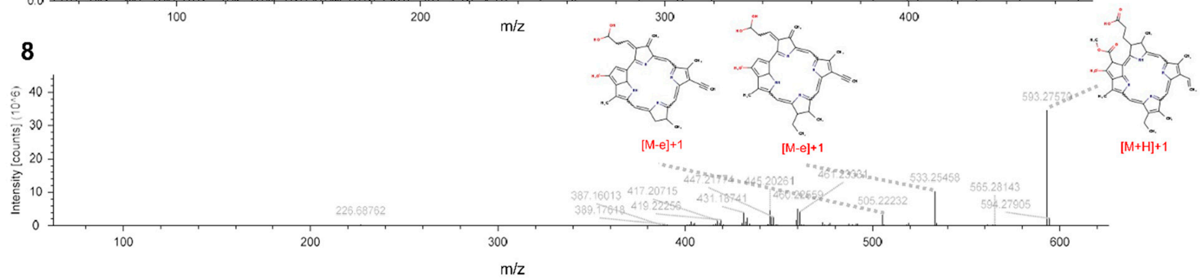
[M-e]+1
[M-e]+1
[M+H]+1
Intensity [counts] (10^6)
m/z
533.25458
565.28143
226.68762

9

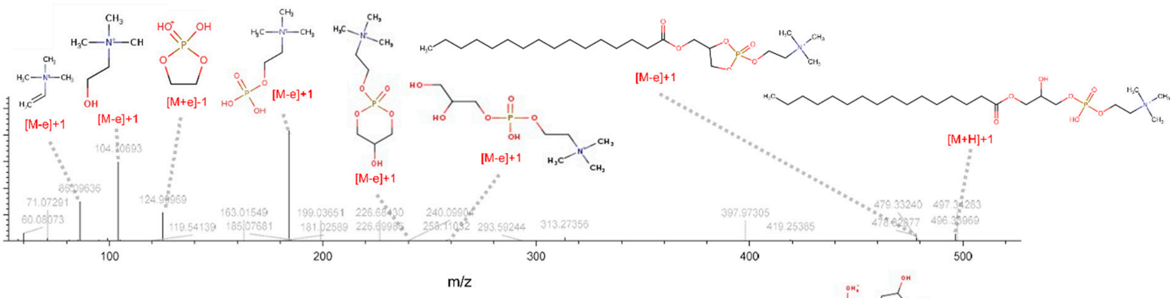

[M-e]+1
[M-e]+1
[M+e]-1
[M-e]+1
[M-e]+1
[M-e]+1
[M-e]+1
[M+H]+1
Intensity [counts] (10^6)
m/z
104.10693
86.09636
71.07291
60.08073
124.99969
119.54139
163.01549
185.07681
199.03651
181.02589
226.68430
240.09904
258.11032
293.59244
313.27356
397.97305
419.25385
479.33240
497.34263
496.33969

10

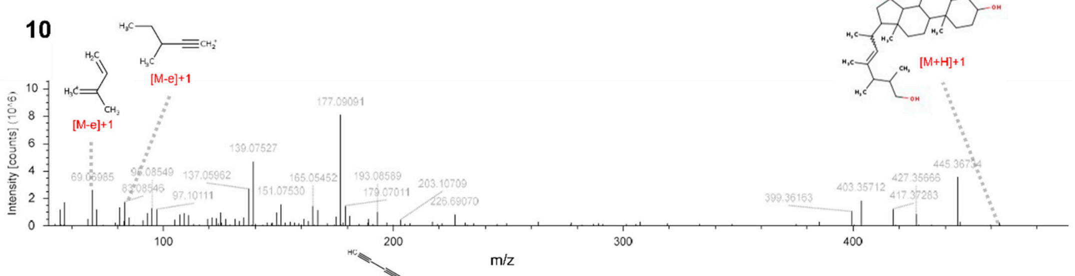

[M-e]+1
[M-e]+1
[M+H]+1
Intensity [counts] (10^6)
m/z
177.09091
139.07527
137.05962
151.07530
165.05452
193.08589
179.07011
203.10709
226.69070
97.10111
399.36163
403.35712
427.35666
417.37283
445.36784

11

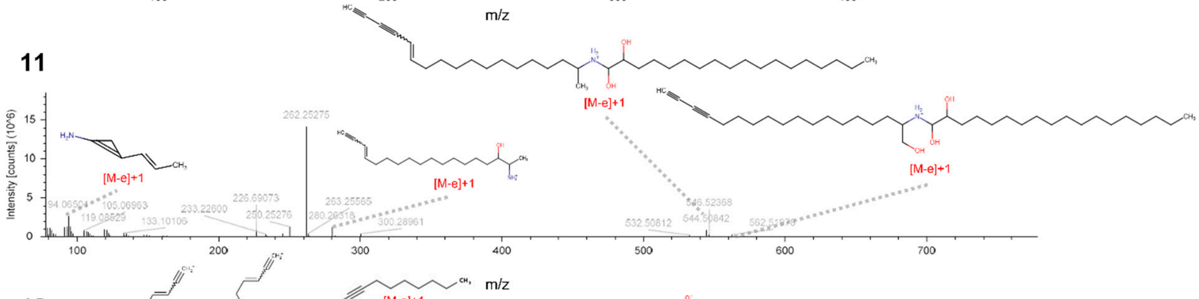

[M-e]+1
[M-e]+1
[M-e]+1
[M-e]+1
Intensity [counts] (10^6)
m/z
262.25275
105.06963
119.08529
133.10106
233.22800
226.69073
250.25276
263.25565
280.26318
300.28961
532.50812
546.52368
544.50842

12

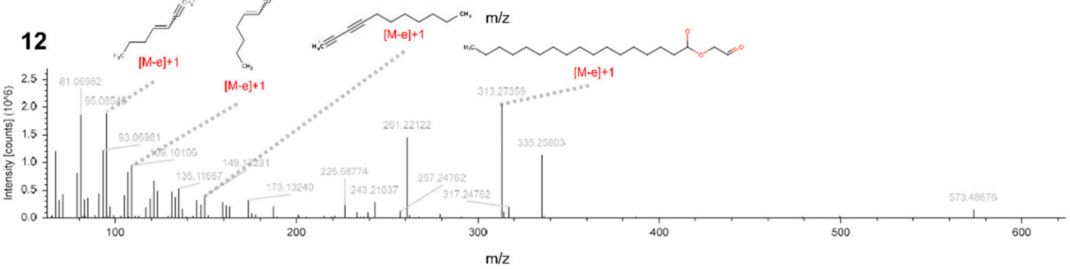

[M-e]+1
[M-e]+1
[M-e]+1
[M-e]+1
Intensity [counts] (10^6)
m/z
81.06982
93.06981
135.11667
149.13231
173.13240
226.68774
243.21037
261.22122
257.24762
317.24762
313.27359
335.25803
573.48676

13

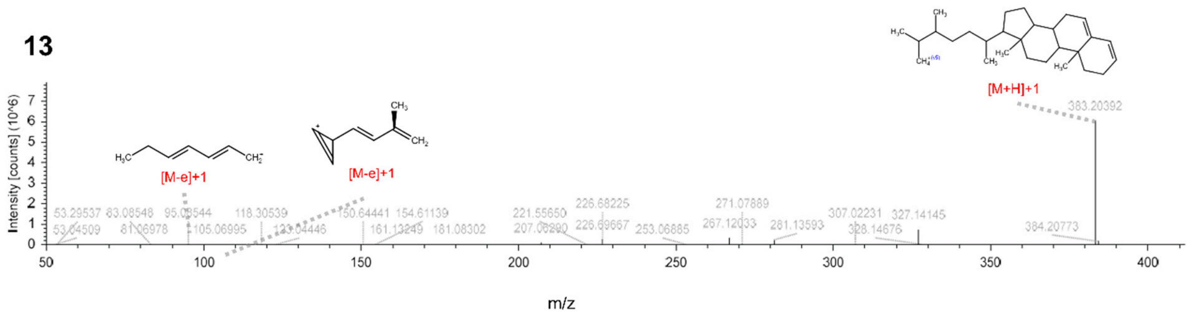

[M-e]+1
[M-e]+1
[M+H]+1
383.20392
384.20773
327.14145
Intensity [counts] (10^6)
m/z

14

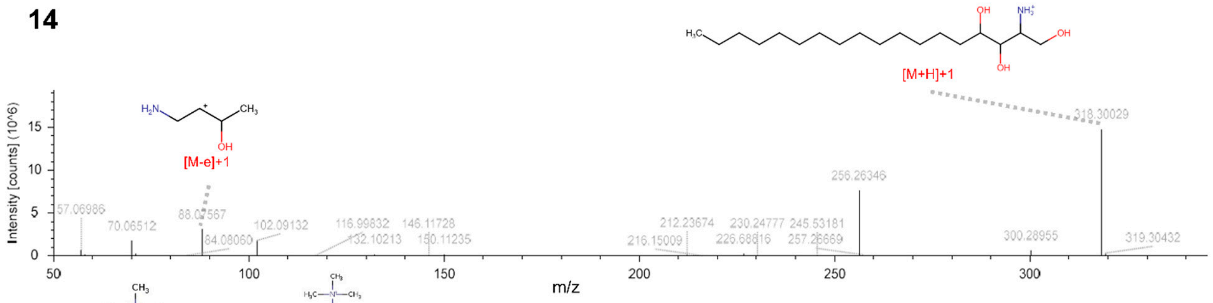

[M-e]+1
[M+H]+1
318.30029
256.26346
319.30432
300.28955
Intensity [counts] (10^6)
m/z

15

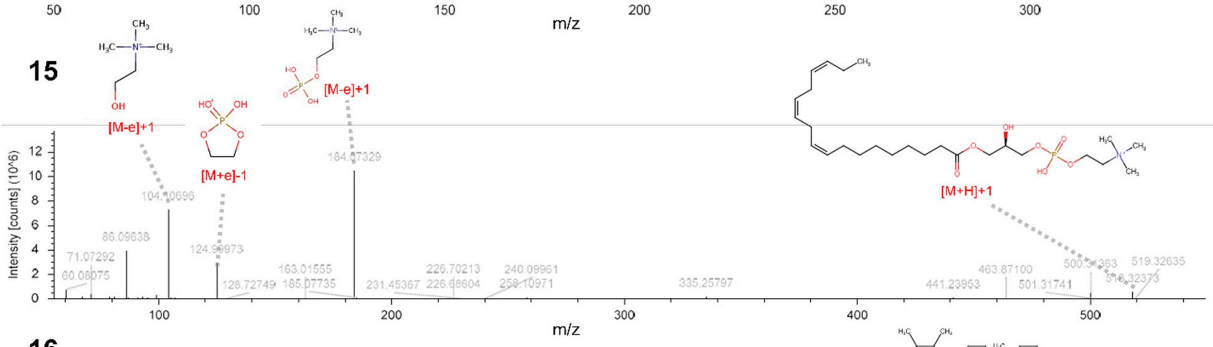

[M-e]+1
[M+e]-1
[M-e]+1
[M+H]+1
184.07329
124.99973
86.09638
Intensity [counts] (10^6)
m/z

16

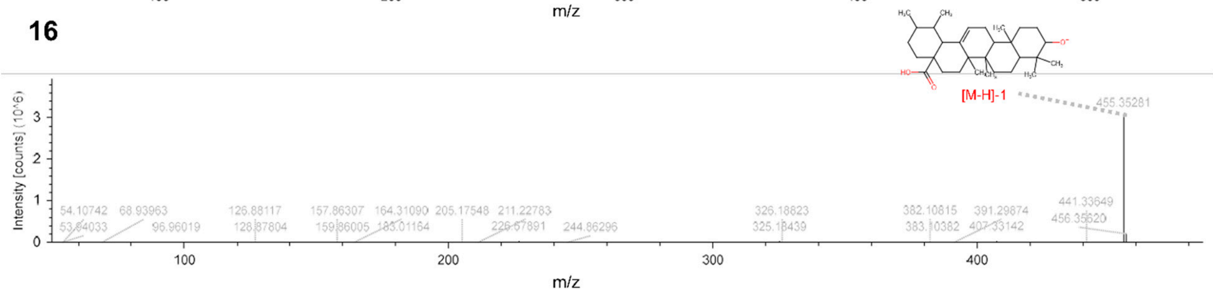

[M-H]-1
455.35281
Intensity [counts] (10^6)
m/z

17

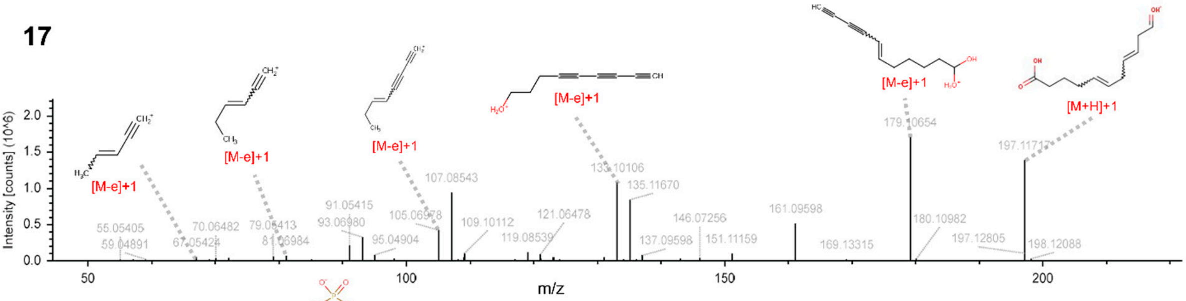
[M-e]+1
[M-e]+1
[M-e]+1
[M-e]+1
[M-e]+1
[M+H]+1
Intensity [counts] (10^6)
55.05405
59.04891
67.05424
70.06482
79.05413
81.06984
91.05415
93.06980
95.04904
105.06978
107.08543
109.10112
119.08539
121.06478
133.10106
135.11670
137.09598
146.07256
151.11159
161.09598
169.13315
179.10654
180.10982
197.12805
197.11717
198.12088
m/z

18

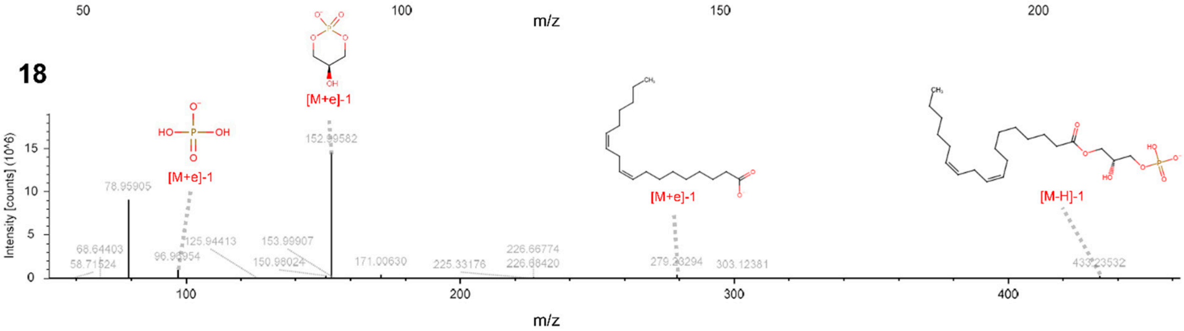
[M+e]-1
[M+e]-1
[M+e]-1
[M-H]-1
Intensity [counts] (10^6)
58.71524
68.64403
78.95905
96.96954
125.94413
150.98024
152.99582
153.99907
171.00630
225.33176
226.66774
226.68420
279.23294
303.12381
433.23532
m/z

19

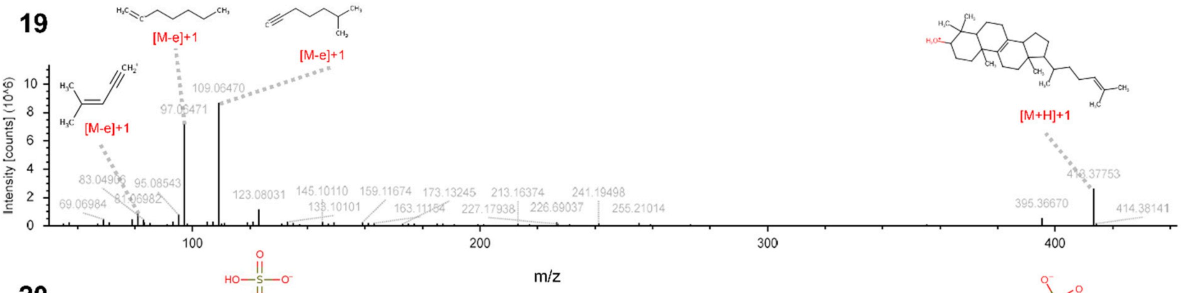
[M-e]+1
[M-e]+1
[M-e]+1
[M+H]+1
Intensity [counts] (10^6)
69.06984
83.04906
81.06982
95.08543
97.08471
109.06470
123.08031
133.10101
145.10110
159.11674
163.11184
173.13245
213.16374
227.17938
226.69037
241.19498
255.21014
395.36670
413.37753
414.38141
m/z

20

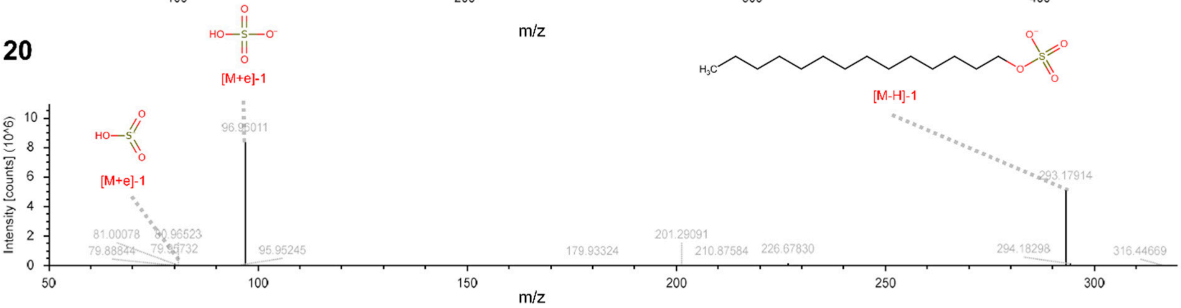
[M+e]-1
[M+e]-1
[M-H]-1
Intensity [counts] (10^6)
81.00078
79.88844
80.96523
79.95732
96.95011
95.95245
179.93324
201.29091
210.87584
226.67830
293.17914
294.18298
316.44669
m/z

21

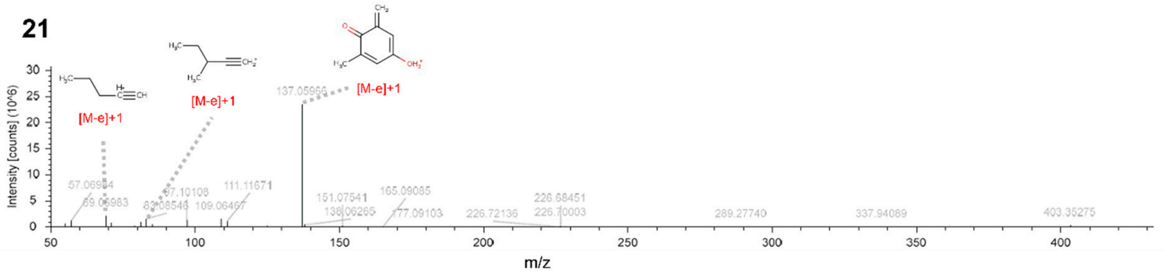

[M-e]+1
[M-e]+1
[M-e]+1
Intensity [counts] (10^6)
137.05966
57.06984
69.06983
97.10108
111.11571
109.06467
151.07541
138.06265
165.09085
177.09103
226.72136
226.68451
226.70003
289.27740
337.94089
403.35275
m/z

22

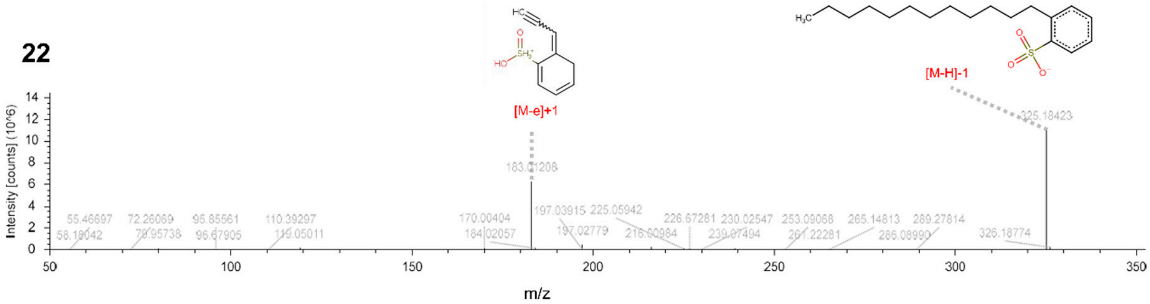

[M-e]+1
[M-H]-1
Intensity [counts] (10^6)
325.18423
183.01208
55.46697
58.18042
72.26069
79.95738
95.85561
96.67905
110.39297
119.05011
170.00404
184.02057
197.03915
197.02779
225.05942
216.00984
226.67281
230.02547
239.07494
253.09068
261.22281
265.14813
286.08990
289.27814
326.18774
m/z

23

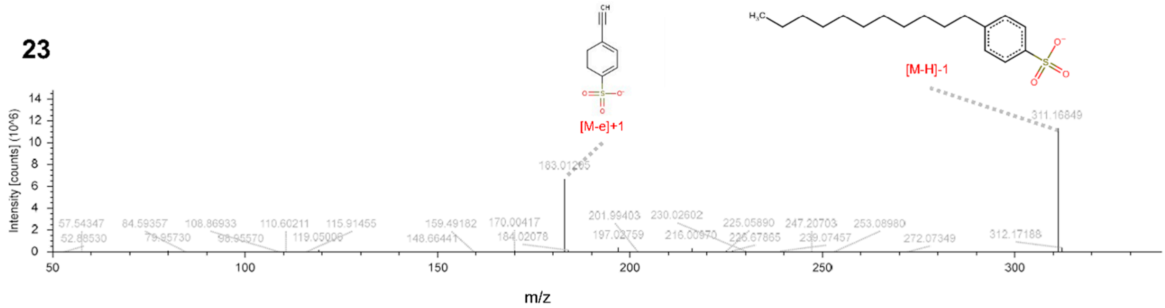

[M-e]+1
[M-H]-1
Intensity [counts] (10^6)
311.16849
183.01265
57.54347
52.68530
84.59357
79.95730
108.86933
98.95570
110.60211
115.91455
119.05006
159.49182
148.66441
170.00417
184.02078
201.99403
197.02759
230.02602
216.00970
225.05890
226.67865
247.20703
239.07457
253.08980
272.07349
312.17188
m/z

Supplementary Figure S5. IC_{50} measurement after treatment with *P. urinaria*, (a) HeLa, (b) HaCaT

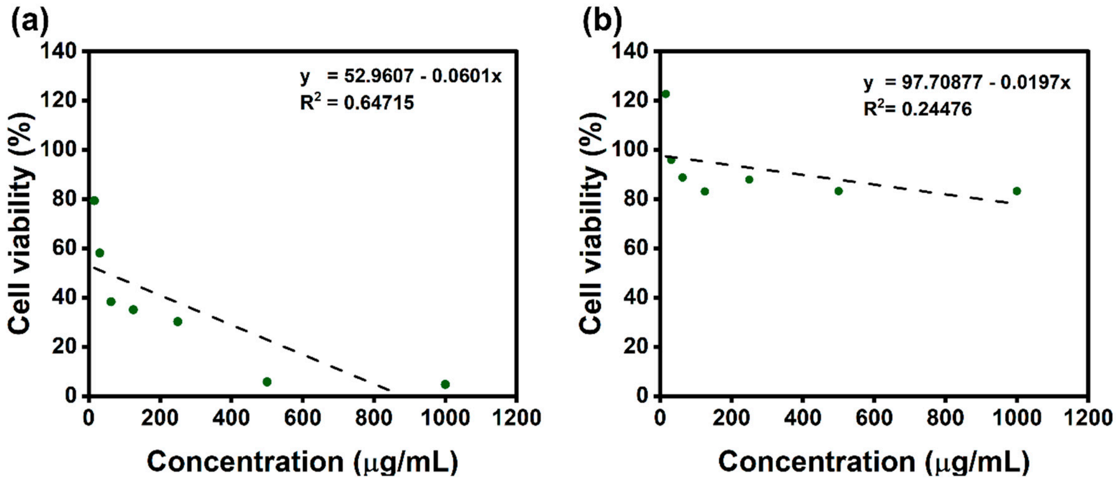

Supplementary Table S1. Annotation of compounds detected by LC-HRMS based on MS^1 mass data

Compound ID	Observed m/z	Annotation delta mass (ppm)	MS level	Adduct	Suggested compound	Chemical formula	Best match
1	613.4821	-0.90	MS^1	[M+H]+1	DG(18:3(9Z,12Z,15Z)/18:3(9Z,12Z,15Z)/0:0)	$C_{39}H_{64}O_5$	Chemspider
2	353.2685	-0.48	MS^1	[M+H]+1	Ceriporic acid C	$C_{21}H_{36}O_4$	Chemspider
3	293.2473	-0.74	MS^1	[M+H+MeOH]+1	6-[1]-ladderane hexanol	$C_{18}H_{28}O$	Chemspider
4	623.2861	-0.55	MS^1	[M+H]+1	2,5-Bis[(2-acetamidobenzoyl)amino]-1,2,5,6-tetradeoxy-1,6-diphenyl-L-altritol	$C_{36}H_{38}N_4O_6$	Chemspider
5	551.4247	-0.11	MS^1	[M+H]+1	Echinenone	$C_{40}H_{54}O$	Chemspider
6	288.2896	-0.22	MS^1	[M+H]+1	Isoheptadecasphinganine	$C_{17}H_{37}NO_2$	Chemspider
7	445.36746	-0.37	MS^1	[M+H]+1	3β-Hydroxy-4β-methyl-5α-cholest-7-ene-4α-carboxylate	$C_{29}H_{48}O_3$	Chemspider
8	593.2757	-0.15	MS^1	[M+H]+1	Pheophorbide A	$C_{35}H_{36}N_4O_5$	Chemspider

9	496.3396	-0.39	MS[1]	[M+H]+1	1-Palmitoylglycerophosphocholine	$C_{24}H_{50}NO_7P$	Chemspider
10	445.36731	-0.38	MS[1]	[M+H-H2O]+1	Certonardosterol J	$C_{29}H_{50}O_4$	Chemspider
11	742.58246	-0.44	MS[1]	[M+H]+1	Dracontioside A	$C_{42}H_{79}NO_9$	Chemspider
12	591.4980	-0.42	MS[1]	[M+H]+1	DG(17:1(9Z)/17:2(9Z,12Z)/0:0)[iso2]	$C_{37}H_{66}O_5$	Chemspider
13	383.36716	-0,19	MS[1]	[M+H]+1	Ergosta-3,5-diene	$C_{28}H_{46}$	Chemspider
14	318.30026	-0,03	MS[1]	[M+H]+1	2-Amino-1,3,4-octadecanetriol	$C_{18}H_{39}NO_3$	MzCloud (81.3%)
15	518.32385	-0,51	MS[1]	[M+H]+1	PC(18:3(9Z,12Z,15Z)/0:0)	$C_{26}H_{48}NO_7P$	Chemspider
16	439.35675	-0,66	MS[1]	[M+H-H2O]+1	Ursolic acid	$C_{30}H_{48}O_3$	MzCloud (97%)
17	197.1171	-0,65	MS[1]	[M+H]+1	11-oxo-undeca-5,8-dienoic acid	$C_{11}H_{16}O_3$	Chemspider
18	433.23574	-0,59	MS[1]	[M-H]-1	PA(18:2(9Z,12Z)/0:0)	$C_{21}H_{39}O_7P$	Chemspider
19	413.37772	-0,19	MS[1]	[M+H]+1	4,4-dimethyl-5a-cholesta-8,24-dien-3-b-ol	$C_{29}H_{48}O$	Chemspider
20	293.17929	0,18	MS[1]	[M-H]-1	Myristyl sulfate	$C_{14}H_{30}O_4S$	Chemspider

21	403.35684	-0,52	MS[1]	[M+H]+1	D-δ-Tocopherol	$C_{27}H_{46}O_2$	MzCloud (97.6%)
22	325.1843	0,02	MS[1]	[M-H]-1	2-Dodecylbenzenesulfonic acid	$C_{18}H_{30}O_3S$	Chemspider
23	311.16867	0,10	MS[1]	[M-H]-1	4-Undecylbenzenesulfonic acid	$C_{17}H_{28}O_3S$	Chemspider

Supplementary Table S2. Molecular interactions of E6 and *P. urinaria.*

Compound	Interaction		
	Hydrogen	Van der Waals	Hydrophobic
(24ξ)-Ergosta-3,5-diene	-	Gly137, Phe52, Cys58, Tyr77, Ser78, Gln114, Ile106	Arg138, Leu107, Arg109, Leu57, Tyr39, Val69, Val 37, Val60, Leu74
[STdimethyl(20)]4_4-dimethyl-cholesta-8_24-dien-3beta-ol	-	Gln114, Phe52, Ala68, Gly137, Trp139, Ile108	Val60, Val38, Tyr39, Val69, Leu74, Arg109, Arg138, Leu107, Cys58, Leu57
1-Palmitoylglycerophosphocholine	Arg109, Arg17	Lys18, Leu107, Gln114, Arg138, Ser78, Ser81, Tyr77, Leu57, Tyr39, Leu74, Val60, Val69, Phe52	-

2,5-Bis[(2-acetamidobenzoyl)amino]-1,2,5,6-tetradeoxy-1,6-diphenyl-L-altritol	Trp139, Arg109	Gly137, Arg138, Thr140, Ile108, Leu108, Leu107, Lys18, Phe52, Leu57, Asp56, Gln114, Ser81, Ser76, Leu74, Val38, Val69	Cys58, Val60, Arg17
2-Amino-1,3,4-octadecanetriol	Cys58, Tyr39	Val38, Val69, Val60, Phe52, Leu74, Gln114, Tyr77, Ile80, Arg84, His85, Ser78, Arg138, Arg109	Leu57

2-Dodecylbenzenesulfonic acid	Cys58	Val60, Val38, Tyr39, Tyr77, Ser78, Gln114, Phe52, Leu57, Trp139, Thr140, Ile108	Leu74, Val69, Leu57, Arg138, Leu107, Arg109
3beta-Hydroxy-4beta-methyl-5alpha-cholest-7-ene-4alpha-carboxylate	Arg138	Arg62, Val38, Val60, Phe52, Lys18, Arg17, Leu107, Gln114	Tyr39, Leu74, Val69, Leu57, Cys58
4-Undecylbenzenesulfonic acid	Ser78, Ser81, Arg138	-	Leu107, Leu57
6-[1]-ladderane hexanol	Cys58	Tyr77, Ser78, Gln114, Ser81, Arg138, Ile135, Leu57, Phe52, Ala68	Leu74, Tyr39, Val38, Val69, Val60

11-oxo-undeca-5,8-dienoic acid	Ser78, Ala68	Phe52, Val60, Val38, Cys58, Leu57, Tyr39, Ser81, Tyr77, Arg138, Gln114	Leu74, Val69
Ceriporic acid C	Arg138, Ser81, Ser78	Tyr77, Gln114, Leu74, Arg109, Arg62, Lys41, Ala68, Phe52, Cys58	Leu57, Val60, Val69, Val38, Tyr39
Certonardosterol J	Tyr77, Arg109, Arg138	Ser81, Ser78, Tyr39, Leu74, Val69, Gln114, Leu57, Lys18, Asp56, Trp139, Arg17	Cys58, Leu107
D-δ-Tocopherol	Gly137, Arg138	Trp139, Ile108,	Leu107, Arg109, Cys58, Leu57,

		Thr140, Asp56, Gln114, Lys18, Leu74, Ala68, Val60, Val38, Arg62	Phe52, Val69, Tyr39
DG(18:3(9Z,12Z,15Z)/18:3(9Z,12Z,15Z)/0:0)	Ser81, His85, Arg84, Tyr39, Tyr77	Ile135, Ile80, Val69, Arg109, Arg17, Lys18, Gln114, Asp56	Arg136, Arg138, Leu74, Leu57, Cys58, Leu107
DG(17:1(9Z)/17:2(9Z,12Z)/0:0)[iso2]	Cys58	Val69, Leu74, Leu57, Gln114, Phe52, Arg136, Ser81, Arg84, Arg62, Val60, Val38, Gly137,	Tyr39, Arg109, Arg138, Leu106, Tyr99, Trp139, Leu107, His85, Tyr77, Ile80

		Lys101, Asp105	
Dracontioside A	Arg109, Tyr39, Cys58,	His85, Arg136, Se81, Ser78, Arg138, Tyr77, Gln114, Leu74, Leu57, Phe52, Leu107, Lys18, Arg17, Asp56	-
Echinenone	-	Trp139, Gly137, Arg109, Gln114, Val69, Val60, Lys41, Lys41	Leu107, Arg138, Leu57, Leu74, Tyr39, Val38, Arg62
Isoheptadecasphinganine	Tyr39	Tyr77, Ser78, Ser81, Gln114,	Cys58, Leu57, Val69

		Arg138, Leu74, Arg109, Asp56, Ala68, Phe52, Val38, Val60	
Myristyl sulfate	Arg138, Ser78, Ser8	Arg62, Arg109, Gln114, Tyr77, Leu74, Phe52	Val38, Val60, Tyr39, Val69, Leu57, Cys58
PA(18:2(9Z,12Z)/0:0)	Arg109, Arg138, Ile108	Gly137, Thr140, Gln114, Ser81, Ser78, Leu74, Phe52, Ala68, Cys58, Leu57, Val38, Tyr39, Arg17, Tyr77	Val60, Val69
PC(18:3(9Z,12Z,15Z)/0:0)	Tyr39, Cys58	Val38, Val60, Leu57,	Arg109, Arg138, Leu107

		Phe52, Val69, Ala68, Leu74, Tyr77, Ser78, Ser81, Gln114, Ile108, Trp139, Thr140, Gly137	
Pheophorbide A	Cys58	Asp56, Arg109, Leu57, Gln114, Ile59, Ala68, Ser78, Tyr77, Ser81	Val60, Val38, Val69, Phe52, Tyr39, Leu74, His85, Arg136
Ursolic acid	His85	Arg136, Cys58, Ala68, Gln114, Leu74, Arg138, Ser78, Tyr77, Ser81	Val38, Val69, Try39, Leu57, Val60, Phe52
